# Examining the Potential Benefits of Affirming Values on Memory for Educational Information

**DOI:** 10.3390/bs15081033

**Published:** 2025-07-30

**Authors:** Karen Arcos, Rebecca Covarrubias, Benjamin C. Storm

**Affiliations:** Department of Psychology, University of California, Santa Cruz, Social Sciences 2, Room 277, Santa Cruz, CA 95064, USA; rgcovarr@ucsc.edu (R.C.); storm@ucsc.edu (B.C.S.)

**Keywords:** learning, memory, value affirmation interventions, first-generation college students, culture, cultural mismatch, independence, interdependence

## Abstract

First-generation students can experience a cultural mismatch between their values and those that colleges and universities tend to prioritize. This mismatch can increase cognitive load, leaving fewer resources available for learning. Effective and long-lasting learning requires actively processing new information and connecting it to existing knowledge—an effort that demands significant cognitive resources. Value affirmation exercises, where students select and reflect upon values that are important to them, have shown promise in reducing cultural mismatch and improving performance on cognitive tasks. However, the impact of these exercises on the learning and recall of new information is less clear. The current study investigated whether a value affirmation exercise, completed before reading an educational passage, would improve memory recall for that passage in a sample of 400 first-generation and continuing-generation young adults, as compared to not affirming. Our results failed to provide evidence that value affirmation exercises impacted recall performance, regardless of whether participants affirmed independent values, interdependent values, or both. Given the importance and implications of this outcome for student learning, we discuss possible explanations for these null findings and suggest future directions in affirmation research.

## 1. Introduction

U.S. institutions of higher education reward cultural values and practices rooted in independence and individualism, such as pursuing individual success and competition (Goudeau et al., 2025; Phillips et al., 2020; Stephens et al., 2012a). Students socialized in contexts that affirm similar values as compared to the university, like middle-to-upper-class contexts, can experience the university as a familiar cultural setting. This cultural match may enable these students to dedicate more of their cognitive resources toward learning new and potentially challenging information. However, students socialized in contexts affirming interdependent values (e.g., connecting to others, valuing cooperation over competition), like working-class contexts, can experience the university’s independent culture as a mismatch (Covarrubias et al., 2019; Phillips et al., 2020; Stephens et al., 2012a; Valle & Covarrubias, 2024; Vasquez-Salgado et al., 2015).

According to cultural mismatch theory, the university norms of independence advantage students from middle-to-upper-class and continuing-generation college backgrounds, while marginalizing students from first-generation college backgrounds, a difference that can lead to disparities in educational outcomes (Phillips et al., 2020; Stephens et al., 2012a). When exposed to the same university message of independence, for example, first-generation students have been shown to cognitively underperform compared to continuing-generation students (Stephens et al., 2012a). Several factors may drive this effect. When first-generation students experience cultural mismatch, they tend to experience increased stress (i.e., cortisol levels), negative emotions, and heightened perceptions of task difficulty compared to what they would have experienced otherwise (Stephens et al., 2012b). Aligning university norms and values with interdependence, however, can ameliorate such effects (Hecht et al., 2021; Tibbetts et al., 2016).

Indeed, cultural motivations for learning—like independent or interdependent motivations—shape what information students pay attention to and/or what they find to be informative. Framing cues from the learning environment to match or align with how learners perceive their environment can affect how students encode, store, and recall to-be-learned information (Wang, 2009). These cultural elements also impact how people conceptualize aspects like space and time, which can in turn affect how events in episodic memory are segmented and ultimately recalled (see Gutchess & Rajaram, 2023; Wang, 2021 for reviews on cultural influences on human memory and cognition).

### 1.1. Signaling a Cultural Match: Value Affirmation Exercises

In contexts where dominant cultural messages persistently mismatch students’ values and motivations, light-touch yet meaningful strategies for signaling a cultural match can support students in their learning and educational outcomes. One such intervention is the use of value affirmation exercises. Value affirmation exercises ask people to choose from a select list of values that are important to them and to reflect on why those values matter to them (Steele, 1988). Across a series of studies, such exercises have been shown to boost performance and feelings of belonging of marginalized students compared to a control condition in which participants select and reflect on values least important to them but important to someone else (Borman et al., 2021; Cohen et al., 2006, 2009; Harackiewicz et al., 2016; Hecht et al., 2021; for a meta-analysis, see Wu et al., 2021). Value affirmation interventions are believed to be effective in part because they enable students to regain or strengthen a sense of self-worth in a context that consistently threatens or stigmatizes aspects of their identities.

A subset of the literature on value affirmation interventions has focused on understanding how independent and/or interdependent values function to affirm students’ identities (Covarrubias et al., 2016; Hoshino-Browne et al., 2005; Tibbetts et al., 2016). For example, one study tested the long-term benefits of a value affirmation intervention in a large introductory biology course (Tibbetts et al., 2016). As part of a class assignment, participants in the intervention condition chose and reflected on independent and interdependent values that were important to them. First-generation students who affirmed their values earned significantly higher grades both that semester and over the following three years compared to first-generation students who did not. Follow-up analyses showed that the extent to which participants wrote about independent values primarily explained this effect. That is, when first-generation students affirmed values that matched the classroom and university culture of independence, their grades improved in subsequent semesters. In this way, value affirmation strategies may function as a pathway for creating a match with university culture.

In a second study, the same authors explored the impacts of affirming independent values on students’ performance on GRE-style math problems (see also Miyake et al., 2010; Tibbetts et al., 2016; Yeager & Walton, 2011). First-generation students who affirmed and reflected on their independent values significantly outperformed first-generation students who affirmed and reflected on their interdependent values. The authors argued that, in affirming values related to independence, students might be able to perceive the learning environment as less socially threatening, thereby allowing them to attend more fully to the challenging cognitive task at hand (Tibbetts et al., 2016).

Importantly, affirming interdependent values has also been shown to benefit marginalized students. For example, when Latinx university students engaged in an interdependent value affirmation exercise (i.e., affirming values important to them and their families), they outperformed Latinx students who engaged in a more independent value affirmation exercise (i.e., affirming values that were individually important to them) on a word puzzle anagram task (Covarrubias et al., 2016). Similarly, other studies have found that women affirming interdependence performed better on math tests, possibly because thinking of their connections with others bolstered their sense of identity and, thus, reduced the cognitive impact that experiencing threat can cause (Shnabel et al., 2013). So, affirming independent or interdependent values within a university setting can both signal a cultural match and, separately, facilitate positive outcomes.

As would be expected, given their distinct capacity to produce positive benefits, affirming both independence and interdependence in a combined value affirmation exercise has also been shown to positively impact academic performance. For example, Tibbetts et al. (2018) found that first-generation students at two-year colleges earned higher course grades when they affirmed both interdependent and independent values compared to a control condition. Moreover, first-generation students from racially minoritized backgrounds (e.g., Latinx, Black) earned higher grades when affirming independence compared to the control condition, whereas the opposite was true for first-generation students from racial majority backgrounds (e.g., White). No differences were found for interdependent-related affirmations. These findings reveal how affirming independence as well as both independence and interdependence can impact learning.

Because the effects and experiences related to cultural mismatch may be less pronounced for participants attending two-year colleges compared to those attending four-year universities (Stephens et al., 2012a; Tibbetts et al., 2016), Hecht et al. (2021) sought to examine the impact of a combined intervention for first- and continuing-generation students attending a four-year university. Again, they found that, compared to a control condition, affirming both interdependence and independence yielded higher scores on a math test and on a cultural match measure among both first- and continuing-generation students. The value affirmation task appeared to reduce participants’ cognitive load and increase confidence amidst stress.

### 1.2. Current Study Aims

In summary, considerable evidence now supports the positive impacts of value affirmation interventions in educational settings. Exactly how such interventions affect learning, however, remains to be fully understood. Moreover, researchers have observed improved performance on specific cognitive tasks (e.g., solving math problems and word puzzles) following an affirmation exercise, as well as in long-term outcomes such as student grade point average. However, little direct research exists on the impact of affirmation exercises on tasks designed to simulate the kind of learning commonly experienced in the classroom. As such, one of the primary goals of the current study was to examine the impact of a value affirmation exercise on students’ ability to read and remember an educational passage.

Research has shown that the ways in which learners engage with to-be-learned information can significantly impact their memory and the extent to which that information becomes integrated with information and knowledge entrenched in long-term memory (e.g., Agarwal & Roediger, 2018; Pan & Bjork, 2024; Rajaram & Barber, 2024). Moreover, learners often employ suboptimal strategies, with students spending substantial time and effort engaging in activities that may feel like they are producing learning even though they are not (Dunlosky et al., 2013). For example, learners often avoid challenging experiences (e.g., Hartwig & Dunlosky, 2012; Kornell & Bjork, 2007), despite the potential of such experiences to enhance long-term learning and transfer (Bjork & Bjork, 2011).

In the context of reading and learning an educational passage, students have significant control in determining the cognitive processes in which they engage. For example, students may or may not retrieve what they had previously read across a given passage to integrate information, thereby synthesizing the content and producing new insights and ideas that further support memory. Likewise, students may or may not interrogate or question what they know, what they are reading, or connect the content to their own interests and existing knowledge. Students who engage in these kinds of processes are much more likely to remember a passage’s contents than those who do not.

Whether students are able to engage in and benefit from these kinds of “desirable difficulties” during self-directed learning is likely to depend on the availability of cognitive resources such as working memory and executive control (e.g., Cortés Pascual et al., 2019). Students who experience cultural mismatch, and other threats related to their identity, may have fewer cognitive resources to engage in the kinds of processes and strategies that benefit learning. If students must allocate cognitive resources towards managing the psychological feelings of stress and negative emotions that cultural mismatch induces, then they may apply fewer cognitive resources towards effectively implementing valuable learning strategies. Similar constraining effects have been demonstrated in a variety of ways in the context of stereotype threat (e.g., Schmader et al., 2008; Szmalec et al., 2011).

Value affirmation exercises may reduce stress and negative emotions, thus mitigating the negative impacts of cultural mismatch. This mitigation allows the cognitive resources that would have been expended towards managing such experiences to be reallocated towards learning. Moreover, value affirmation exercises could encourage learners to engage more deeply with to-be-learned material. In the present study, participants at four-year universities (consisting of both first-generation and continuing-generation students and recent graduates) were randomly assigned to one of four conditions: an independent condition in which they affirmed independent values; an interdependent condition in which they affirmed interdependent values; a combined condition in which they affirmed both independent and interdependent values; or a control condition in which they did not affirm any values. Participants were then asked to read an educational passage and then attempt to recall as much information from the passage as possible.

Unlike past studies’ control conditions, which instructed participants to affirm the values least important to them (Borman et al., 2021; Cohen et al., 2006, 2009; Harackiewicz et al., 2016; Hecht et al., 2021), we utilized a no-value-affirmation control condition. This was because some past studies instructing participants to reflect on values “important to someone else” have inadvertently operated as interdependent affirmations, especially when participants reflect on values important to family (Covarrubias et al., 2016). To avoid the potential for this, we opted to use a no-value-affirmation control condition, in which participants were asked to find anagrams amidst scrambled letters, a task that past value affirmation studies have employed (Covarrubias et al., 2016; Stephens et al., 2012a), albeit as a dependent variable instead of as a control condition. The anagram task was designed to engage participants and require their attention in a way similar to the affirmation conditions without creating stress, threat, or affecting their sense of belonging.

Through this design, we aimed to make at least two contributions. First, to our knowledge, no studies published to date have measured the immediate effects of a value affirmation exercise on participants’ recall of newly learned information. Second, by including a combined condition, we sought to better tease apart the singular and additive effects of independent and interdependent affirmations for students attending or who have attended a four-year university. Based on the research reviewed above, we expected first-generation participants to benefit from conditions that involved affirming independent values (Miyake et al., 2010; Tibbetts et al., 2016; Yeager & Walton, 2011), as well as conditions that involved affirming interdependent values (Covarrubias et al., 2016; Hoshino-Browne et al., 2005), perhaps accruing the greatest benefits when affirming both (Tibbetts et al., 2018). Owing to the lack of pre-existing cultural mismatch, we expected the affirmation intervention to affect continuing-generation students relatively less. More generally, the study provides an important step towards better understanding whether and how different kinds of value affirmation interventions have the potential to impact learning.

## 2. Methods

### 2.1. Participants

Prior to recruitment, we conducted a power analysis to gain the thresholds for 80% power. Each condition needed 100 participants to achieve a Cohen’s *d* of 0.40 between any two conditions. Thus, we aimed to recruit 400 participants and stopped data collection as soon as that target was met. This sample size was chosen in an effort to best balance our resource constraints and desire to have enough power to detect a small to medium effect size that would be both practically and theoretically informative.

A total of 400 participants completed the study (*Mean* Age = 23.11; *SD* = 2.63; range = 18 to 34 years). Participants were recruited using Prolific software (Prolific, London, UK), 1 November–1 December 2022. Participants were eligible if they were 18–27 years of age (four participants were included despite being older than the maximum age), resided in the U.S., and were either current undergraduate students or alumni who had recently earned their undergraduate degrees from four-year institutions. Students from all majors were eligible. To recruit participants, we prescreened for age, English fluency, and an approval rate on Prolific of 80–100%. We further prescreened for participants’ highest education level earned to determine current and recent undergraduate status, as well as highest parental education level to determine college generation status. We excluded one participant for failing to pass an attention check, bringing the final sample to 399. Table 1 details demographic information focusing specifically on the 391 participants for whom we were able to determine college generation status. Analyses not including college generation status included data from the entire set of 399 participants.

We chose to include recent graduates, and not just current undergraduate students, because it allowed us to recruit from a larger and more diverse population. Moreover, most studies to date have not examined these effects in recent graduates, and there is good reason to think that cultural mismatch experiences extend post-graduation, especially within the context of U.S. professional workplaces (e.g., Stephens et al., 2019).

### 2.2. Procedure, Materials, and Measures

Participants were randomly assigned to and completed one of four value affirmation conditions: independent (n = 102), interdependent (n = 105), combined (n = 115), and control (n = 77). It is unclear why fewer participants were run in the control condition than in the other conditions, but it may have been due to an issue with recruiting in Prolific. Importantly, however, participants were randomly assigned to the conditions. We thus have no reason to think that the participants in the four conditions had pre-existing differences. After completing the value affirmation exercise (or not), all participants read an educational passage at their own pace and were then immediately tested on their memory of the contents of that passage. Finally, all participants completed two rating scales, were debriefed on the study’s purpose, and were compensated USD 10 for participating. The entire study took about 30 min to complete per participant. All study procedures and materials were approved as exempt by the UC Santa Cruz Institutional Review Board #HS-FY2022-372.

#### 2.2.1. Value Affirmation Manipulation

The value affirmation conditions were modeled after the designs employed by Tibbetts et al. (2016) and Hecht et al. (2021). Specifically, we selected three values associated with interdependence (i.e., Relationships with Friends and Family, Spiritual or Religious Values, and Belonging to a Social Group), three values associated with independence (i.e., Independence, Learning and Gaining Knowledge, and Curiosity), and two neutral values that were neither highly associated with interdependence nor independence (i.e., Being Good at Art, Government and Politics). The interdependent and independent values have been used in past research, and past participants have been shown to commonly select and write about them (Harackiewicz et al., 2016). The neutral values have also been used in past research, but participants have tended to select them least. The neutral values were included as alternatives to create an illusion of choice while still encouraging participants to write about independence and interdependence (Tibbetts et al., 2016). The value affirmation prompts are available on the Open Science Framework (OSF).

Independent condition: Participants were presented with five values (three related to independence: Independence, Learning and Gaining Knowledge, Curiosity; two neutral: Being Good at Art, Government and Politics) and asked to select the two values that were most important to them. Once the two values were selected, participants were asked to write two paragraphs (4–5 sentences each) explaining why each value was important to them.

Interdependent condition: Participants were presented with five values (three related to interdependence: Relationships with Friends and Family, Spiritual or Religious Values, Belonging to a Social Group; two neutral: Being Good at Art, Government and Politics) and asked to select the two values that were the most important to them. Once the two values were selected, participants were asked to write two paragraphs (4–5 sentences each) explaining why each value was important to them.

Combined condition: Participants were presented with four values (two related to independence: Learning and Gaining Knowledge, Curiosity; two related to interdependence: Relationships with Friends and Family, Belonging to a Social Group) and asked to select the two values that were most important to them. More specifically, they were asked to select one value related to independence, and one value related to interdependence. Neutral values were excluded from this condition to ensure that participants reflected on both independence and interdependence. The instructions for writing two paragraphs mirrored the other conditions.

Control condition: Participants did not engage in any form of value affirmation exercise. Although prior research has sometimes employed a control condition in which participants write about values that are not important to them but are important to others, such an activity could inadvertently act as a kind of value affirmation exercise, especially if participants were prompted to reflect on values important to their family members. Thus, we asked participants to complete an anagram task instead. Participants rearranged nine scrambled letters into as many meaningful 4 to 6 letter words as possible, with 44 words to find. Participants identified, on average, less than a fifth (*M* = 0.16, *SD* = 0.10) of all the words possible, and did not receive feedback.

Two independent raters scored each value affirmation response for the absence (scored as 0) or presence (scored as 1) of independent and interdependent themes (see Hecht et al., 2021, Appendix B for scoring criteria). To be coded for independence, at least one of the following criteria needed to be met: expressing the value of independence for the self, valuing an activity because it is done alone, and showing that the participant values their own autonomy. To be scored as interdependent, the criteria included valuing an activity because it is done with others, feeling like part of a group of people because of a certain value or while engaging in a certain activity, and mentioning thoughts of interdependence. A given response could receive scores for both independence and interdependence. Our inter-rater reliability on coding was near-perfect for independence (Cohen’s kappa = 0.99; Landis & Koch, 1977), and perfect for interdependence (Cohen’s kappa = 1.00).

Through this analysis, we were able to confirm that participants expressed values generally as assigned by their condition. Participants in the independent condition were much more likely to express values related to independence (*M* = 0.96) than interdependence (*M* = 0.08), and participants in the interdependent condition were much more likely to express values related to interdependence (*M* = 0.82) than independence (*M* = 0.29). Participants in the combined condition showed a slight bias towards expressing values related to independence (*M* = 0.60) over independence (*M* = 0.45).

A total of 18 participants (2, 2, and 14 in the independent, interdependent, and combined conditions, respectively) did not follow the instructions exactly as directed, either by writing paragraphs that appeared to be off-topic or by clearly focusing on values other than those to which they were assigned. This was particularly common in the combined condition, with participants continuing to focus on the first value in their second paragraph instead of switching to the second value. For the purposes of ensuring internal validity, we opted to include these participants in their assigned conditions (i.e., reassigning participants to other conditions could create selection effects, thus leading to pre-existing differences between groups in the four conditions). Moreover, all participants experienced the affirmation intervention as assigned, and so any variance in how the participants responded to the intervention was important to include when considering potential applied implications.

#### 2.2.2. Learning and Recalling Information from an Educational Passage

To examine the potential effects of the value affirmation exercises on learning, we used an educational passage about the science of ground water. The passage was 856 words long and taken from the U.S. Geological Survey website (Clark & Briar, 1993). Participants were instructed to read the passage at their own pace and were told that they would be tested on their ability to recall information from the passage. They were also told that the passage was unrelated to the exercises completed earlier. After reading the passage, the participants were immediately asked to type out as much information as they could recall from the passage in any order and not to worry about spelling or grammar. Participants were given as much time as they needed to complete the recall task, opting to end the task when they felt they could not recall any additional information.

For the purpose of scoring recall performance, a total of 52 idea units were identified in the passage. Two raters read each participant’s recall output and determined whether each idea unit was addressed in their output. Credit was given for idea units even if they were only partially addressed, regardless of word order, spelling, or grammar. Credit was not given for idea units that were either not addressed or that were addressed incorrectly. To calculate recall performance, the number of retrieved idea units was divided by 52 for each participant. Inter-rater reliability was perfect between the two raters (intraclass correlation coefficient = 1.00). Any disagreements in the scoring of individual items were resolved by a third rater prior to analysis.

#### 2.2.3. Additional Measures

Two measures were administered after completing the free-recall task: the Cultural Congruity Scale (Gloria & Kurpius, 1996) and the Independent and Interdependent Motivations Scale (Stephens et al., 2012a). We hoped to use the responses on these scales to better understand how individual differences in cultural congruity and motivations to attend college might affect the impact of value affirmation exercises. Upon further reflection, however, we opted to focus on independent and interdependent motivations. Although cultural congruity can be useful to measure in many contexts, it is not strongly aligned with how Stephens et al. (2012a) define cultural mismatch (i.e., the clash between independence and interdependence) and, thus, may tap into something different than what we had hoped to measure.

The Independent and Interdependent Motivations Scale includes 12 items that assess the extent to which participants endorse independent or interdependent motives for attending college from 1 (strongly disagree) to 6 (strongly agree) (Stephens et al., 2012a). The scale consists of six independent items (e.g., Expand my knowledge of the world) and six interdependent items (e.g., Help my family out after I’m done with college). The items were averaged to create two subscales, with higher scores indicating higher independent (Cronbach’s alpha = 0.87, *Mean* = 5.08, *SD* = 0.74) and interdependent (Cronbach’s alpha = 0.82, *Mean* = 3.98, *SD* = 1.10) motivations.

## 3. Results

We first examined recall performance on the ground water passage as a function of college generation status and value affirmation condition. Specifically, we employed a 2 (first-generation vs. continuing-generation) × 4 (independent vs. interdependent vs. combined vs. control) between-subjects ANOVA, with the proportion of idea units (out of 52) included as the dependent variable. Neither of the main effects were statistically significant. Specifically, recall failed to differ significantly as a function of college generation status (first-generation: *M* = 29.5%, *SE* = 1.1%; continuing-generation: *M* = 28.5%, *SE* = 1.0%)—*F*(1, 383) = 0.46, *MSE* = 0.02, *p* = 0.50, ηp^2^ = 0.00—or Value Affirmation Condition (independent: *M* = 30.7%, *SE* = 1.5%; interdependent: *M* = 27.6%, *SE* = 1.4%; combined: *M* = 29.1%, *SE* = 1.4%; control: *M* = 28.6%, *SE* = 1.7%)—*F*(3, 383) = 0.82, *MSE* = 0.02, *p* = 0.48, ηp^2^ = 0.01.

**Figure 1:**
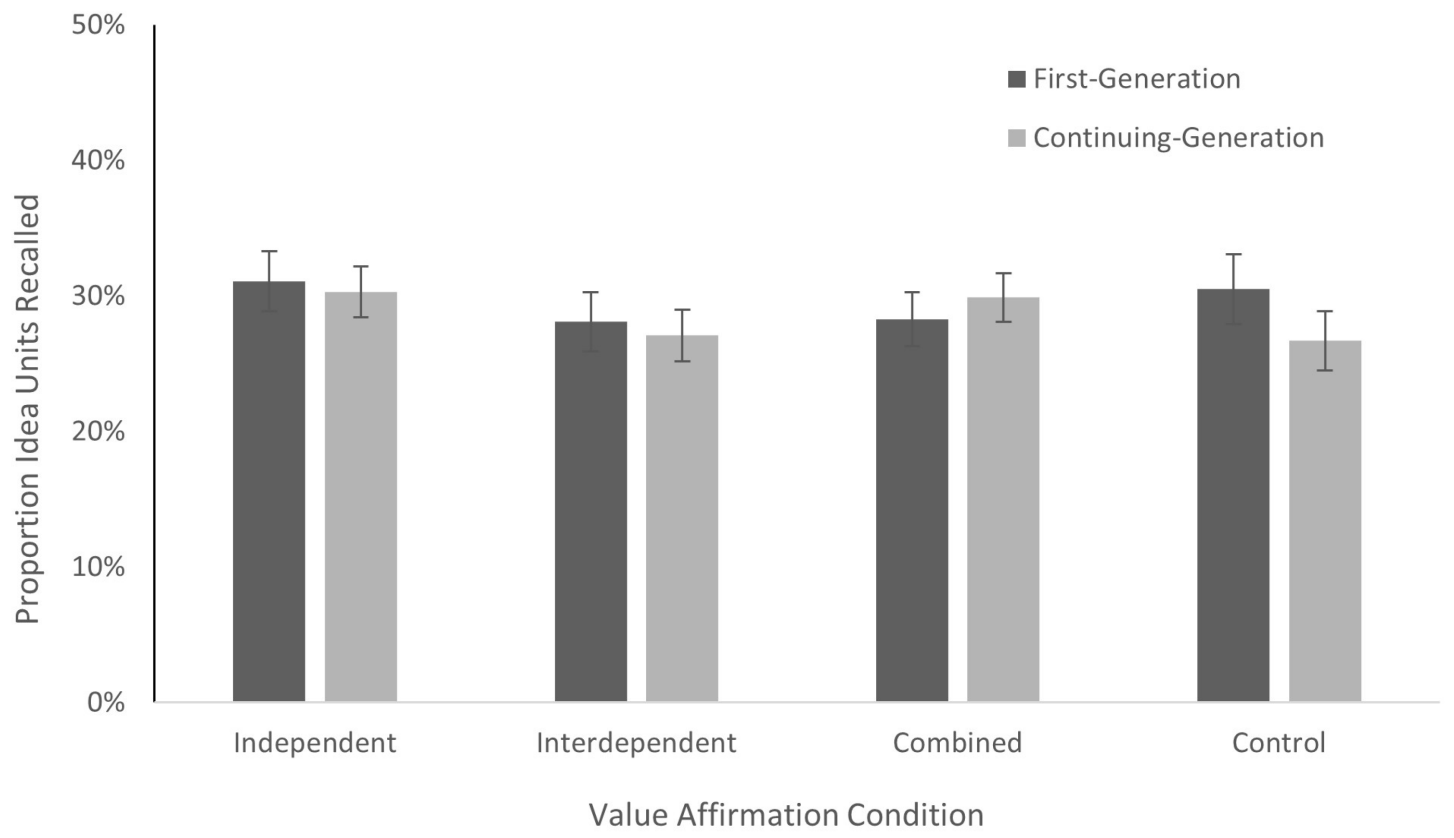
Value affirmation conditions by college generation status on units recalled. Note. Recall performance is shown for the four conditions as a function of college generation status. The error bars show the standard errors of the mean.

As shown in Figure 1, we also failed to find evidence of a significant interaction between the two factors: *F*(3, 383) = 0.54, *MSE* = 0.02, *p* = 0.66, ηp^2^ = 0.00. Recall performance was approximately the same across value affirmation conditions for both the first-generation participants (independent: *M* = 31.1%, *SE* = 2.2%; interdependent: *M* = 28.1%, *SE* = 2.2%; combined: *M* = 28.3%, *SE* = 2.0%; control: *M* = 30.5%, *SE* = 2.6%) and continuing-generation participants (independent: *M* = 30.3%, *SE* = 1.9%; interdependent: *M* = 27.1%, *SE* = 1.9%; combined: *M* = 29.9%, *SE* = 1.8%; control: *M* = 26.7%, *SE* = 2.2%). Including age, gender, ethnicity, and educational status as covariates did not change the observed pattern of results.

We conducted several follow-up analyses to confirm the robustness of these results. None of these analyses were pre-registered. First, we ran a one-way ANOVA including all participants (not just those for whom we were able to determine college generation status). The results were the same, with no evidence of a significant effect of Value Affirmation Condition: *F*(3, 395) = 0.85, *MSE* = 0.02, *p* = 0.47, ηp^2^ = 0.01. Then, we re-ran the 2 × 4 ANOVA reported above while excluding the 18 participants who did not complete the value affirmation task as instructed. These participants either wrote essays that were off-topic or did not focus on the particular values assigned. The pattern of results was once again the same, with no evidence of a main effect of college generation status—*F*(1, 365) = 0.56, *MSE* = 0.02, *p* = 0.45, ηp^2^ = 0.00—or value affirmation condition—*F*(3, 365) = 1.02, *MSE* = 0.02, *p* = 0.39, ηp^2^ = 0.01—or an interaction between the two factors—*F*(3, 365) = 0.39, *MSE* = 0.02, *p* = 0.76, ηp^2^ = 0.00.

Finally, to provide a more sensitive measure of the potential benefits of engaging in any value affirmation exercise, we collapsed all the participants who passed the attention check across the three value affirmation conditions into a single category. A 2 (first-generation vs. continuing-generation) × 2 (value affirmation vs. control) ANOVA once again failed to find any significant main effect of college generation status—*F*(1, 387) = 1.04, *MSE* = 0.02, *p* = 0.31, ηp^2^ = 0.00—or Value Affirmation Condition—*F*(1, 387) = 0.06, *MSE* = 0.02, *p* = 0.80, ηp^2^ = 0.00—or an interaction between the two factors—*F*(1, 387) = 0.104, *MSE* = 0.02, *p* = 0.31, ηp^2^ = 0.00. Indeed, neither first-generation participants (value affirmation: *M* = 29.1%, *SE* = 1.2%; control: *M* = 30.5%, *SE* = 2.6%; *t*(167) = −0.52, *p* = 0.61, *d* = −0.10, 95% CI = [−0.49, 0.29]) nor continuing-generation participants (value affirmation: *M* = 29.1%, *SE* = 1.1%; control: *M* = 26.7%, *SE* = 2.2%; *t*(220) = 0.96, *p* = 0.34, *d* = 0.16, 95% CI = [−0.17, 0.50]) recalled significantly more idea units in the collapsed value affirmation conditions than they did in the control condition.

The results reported above failed to provide statistically significant evidence of the value affirmation exercises impacting recall performance. To better quantify evidence for the null, three Bayesian independent-samples *t*-tests were performed, comparing each of the value affirmation conditions with the control condition. All three Bayes Factors suggested moderate evidence for the null over the alternative (independent: BF = 5.8; interdependent: BF = 7.1; combined: BF = 8.1). A fourth analysis comparing the combination of the three affirmation conditions with the control condition also provided moderate evidence for the null, BF = 9.7. To give some context, a BF of 9.7 suggests that the data are approximately 9.7 times more likely under the null hypothesis than under the alternative hypothesis.

It is important to note that we failed to observe any significant differences in independent motivations (first-generation: *M* = 5.11, *SE* = 0.06; continuing-generation: *M* = 5.06, *SE* = 0.05) and interdependent motivations (first-generation: *M* = 4.07, *SE* = 0.09; continuing-generation: *M* = 3.91, *SE* = 0.07) as a function of generation status. Thus, to try to more directly examine the effect of motivations, we split the participants into two groups based on their responses to the Independent and Interdependent Motivations Scale. Specifically, we subtracted participants’ independent motives score from their interdependent motives score to calculate a difference score for each participant and then rank-ordered the difference scores. A median split was used to create two groups of participants: one with relatively high interdependent motivations (independent motivations: *M* = 4.86, *SD* = 0.83; interdependent motivations: *M* = 4.75, *SD* = 0.75), and one with relatively high independent motivations (independent motivations: *M* = 5.29, *SD* = 0.57; interdependent motivations: *M* = 3.26, *SD* = 0.87). We then ran a 2 (interdependent motivations vs. independent motivations) × 4 (independent vs. interdependent vs. combined vs. control) between-subjects ANOVA, and as in the analysis reported above, no evidence of a significant interaction was observed: *F*(3, 391) = 0.59, *MSE* = 0.02, *p* = 0.62, ηp^2^ = 0.01. Recall performance was approximately the same across the value affirmation conditions both for participants with relatively high interdependent motivations (independent: *M* = 28.4%, *SE* = 2.2%; interdependent: *M* = 26.8%, *SE* = 2.0%; combined: *M* = 26.6%, *SE* = 1.9%; control: *M* = 28.3%, *SE* = 2.2%) and for participants with relatively high independent motivations (independent: *M* = 32.2%, *SE* = 1.9%; interdependent: *M* = 28.81%, *SE* = 2.0%; combined: *M* = 32.2%, *SE* = 2.2%; control: *M* = 29.1%, *SE* = 2.4%).

## 4. General Discussion

Given the persisting mismatch that many first-generation students navigate in university contexts, understanding which kinds of values to affirm offers a pathway for creating meaningful cultural alignments for students. We sought to investigate whether affirming for independent values, interdependent values, or both would affect the recall of an educational passage in current and recent first- and continuing-generation undergraduates relative to engaging in a non-affirmation control task. Contrary to past work documenting the separate benefits of independent (Miyake et al., 2010; Tibbetts et al., 2016; Yeager & Walton, 2011) and interdependent (Covarrubias et al., 2016; Hoshino-Browne et al., 2005) affirmations for marginalized students, we failed to find significant impacts of any of our affirmation conditions on recall performance.

Null results can be difficult to interpret. Yet they can also reveal important insights, especially regarding the sensitivity of replicating findings. We find value in pinpointing some of the differences in our study that might have produced different results from earlier work. Indeed, though we closely followed intervention procedures outlined in previous studies (Covarrubias et al., 2016; Hecht et al., 2021), key design differences may partially explain our null effects. We detail these here and discuss possible directions for future research.

One important difference between the current study and most prior research on value affirmation is that the current study was conducted online. Dowell et al. (2021) also failed to observe significant benefits of value affirmation when data were collected online. Some online learning environments may not be conducive to developing particular skills, like reading comprehension, to the same degree as in-person learning (Patronis & Dubai, 2014). More importantly, because many participants likely completed the task at home and away from the university, they may have come into the experiment experiencing relatively less cultural mismatch than they would have in an in-person lab or classroom environment, thus diminishing the potential benefits of the value affirmation intervention. A more sensitive approach may be to focus on contexts in which learners are experiencing greater cultural mismatch to begin with. Still, given that undergraduates are increasingly learning in online environments, which has considerations both for mitigating and exacerbating cultural mismatches (Basch et al., 2022), our design becomes especially well-suited for examining online environments as critical aspects of the student experience. Future research might more directly test differences in performance tasks, including but not limited to the recall of to-be-learned information, across online versus in-person study designs.

Moreover, unlike other affirmation studies that have focused on undergraduate students still enrolled in university settings (Dowell et al., 2021; Hecht et al., 2021; Tibbetts et al., 2016), we included a diverse representation of both current undergraduate students and those who completed their degrees. Though they were diverse in terms of education level, this broader focus, especially by accessing participants with the resources to complete an online study, might also explain why our sample skewed toward more middle-class participants (with 79% reporting earning >USD 60,000 per year). Participants from middle-class backgrounds experience a different cultural mismatch than those from lower-income backgrounds. Moreover, students who have already completed their degree may also experience a different kind of mismatch or identity threat after graduating, especially when encountering the kind of learning task we administered in the current study. In this way, our decision to include college graduates could have contributed to the null effects that were observed. Future investigations with more analytic power might consider how educational status and/or income background inform experiences of a cultural mismatch and, thus, the impacts of the intervention. In fact, an even more direct test of this might include measuring participants’ experiences of cultural mismatch that align with Stephens et al. (2012a)’s conceptualization and how an affirmation treatment speaks to those experiences. Research has offered creative ways of assessing more conceptually aligned and nuanced experiences of cultural mismatch (see Yılmaz et al., 2024), but has yet to assess the link to affirmation strategies.

Another possible interpretation of the current results is that, although the value affirmation exercises were effective in reducing cultural mismatch, the cognitive benefits accrued were not sufficient to enhance performance on the learning task. The cognitive processes involved in studying and recalling information from an educational passage may differ from those operating in prior affirmation tasks, like completing math problems (Covarrubias et al., 2016; Hecht et al., 2021; Shnabel et al., 2013; Tibbetts et al., 2016). Tasks involving processes like multiplying may involve a stronger or more direct working memory component than reading and recalling information and be relatively more likely to elicit identity threat. Thus, value affirmation exercises that enhance working memory may affect the former relatively more. If participants were given specific strategies that depended on employing working memory in the learning task, perhaps a benefit would have been observed. However, the intervention may not have affected the spontaneous strategies participants employed, especially in an online learning environment.

Students often fail to use learning strategies known to boost long-term learning and transfer, choosing instead to engage in activities like rereading or highlighting. Techniques that optimize learning include self-testing, applying read material, and connecting to prior knowledge to practice what has been learned (Bjork & Bjork, 2011; Hartwig & Dunlosky, 2012; Kornell & Bjork, 2007). Without direct instructions on these learning strategies and time to engage with them, values affirmations might not be as likely to impact learning. This is potentially the case when participants engage in reading material on topics they are less familiar with, like the science of ground water. Had participants been better able to connect the content to prior knowledge, especially via a relevant affirmation exercise, they could have created new retrieval pathways to support the learning process and potentially improve recall. Indeed, a subset of affirmation interventions include utility-value affirmations. Their main goal is to signal the usefulness or utility of the presented information, thus creating links to one’s prior knowledge or future goals; these similarly boost performance outcomes for marginalized students (see Harackiewicz et al., 2016). Choosing a topic that better signals this utility might enhance the overall effectiveness of an affirmation, particularly when learning is involved. Another possibility is that one or more of the value affirmation exercises did have a positive impact on how participants studied the passage, but the immediate test was not sensitive to that impact. Work on desirable difficulties has shown that the benefits of many learning interventions can become greater over time (Bjork & Bjork, 2011). Thus, value affirmation exercises might be more likely to enhance performance on a delayed final test than on an immediate final test.

Reporting research regardless of whether significant results are obtained, or prior patterns are replicated, is critically important. Doing so provides a clearer picture of the theoretical mechanisms and boundary conditions of a given phenomenon. The current study was well-powered, with a final sample of 399 participants, while including multiple value affirmation exercises. Although reasons may exist to discount the current null results, the null results should also not be ignored. Future researchers should consider them carefully if attempting to explain when and how value affirmation exercises have the potential to promote learning and academic performance.

Although the current study failed to yield statistically significant results, the benefits of value affirmations in other settings are evident. Investigating the unique effects of independent, interdependent, and combined affirmations under different conditions provides important insights for sharpening their relevancy for students, especially those experiencing strong mismatches within the university. With a more deliberate design, that future work should necessarily explore the impact for something as important and central as learning and recalling new information. Understanding how value affirmation interventions can facilitate learning, including unpacking the conditions and mechanisms by which this happens, has valuable research and teaching implications. Better understanding the mechanisms behind value affirmations’ effects on learning will help to delineate the ways in which we can most effectively apply cognitive psychology principles to support student learning. Such understandings will not only sharpen our knowledge about the range of impacts of affirmations but, if effective, will also serve to strengthen the tools we can employ in the classroom to reduce cultural mismatches and improve student learning.

## Figures and Tables

**Table 1:** Participant demographic information disaggregated by college generation status.

Demographic Category	First-Gen (n = 169)	Continuing-Gen (n = 222)
**Gender**		
Man	45%	45%
Woman	50%	50%
Non-binary, non-conforming, bigender, transgender	5%	5%
**Race/Ethnicity**		
African American, Black, Caribbean	11%	9%
Asian, Asian American	18%	18%
Caucasian, White, European American	42%	62%
Latinx, Hispanic, Chicanx	20%	4%
Middle Eastern	1%	1%
Native American, American Indian, Alaska Native	1%	1%
Mixed Race/Ethnicity	8%	6%
Prefer Not To Respond	0%	0%
**Academic Level**		
Current undergraduate students	48%	51%
Earned undergraduate degree	51%	48%
Unknown	2%	1%

## Data Availability

The data collected and materials employed are available on the Open Science Framework (OSF) project, accessed on 20 February 2025: https://osf.io/zb7kc/?view_only=cd8c0bbd59234310833ac24f711f3789.

## References

[B1-behavsci-15-01033] Agarwal P. K., Roediger H. L. (2018). Lessons for learning: How cognitive psychology informs classroom practice. Phi Delta Kappan.

[B2-behavsci-15-01033] Basch S., Covarrubias R., Wang S.-h. (2022). Minoritized students’ experiences with pandemic-era remote learning inform ways of expanding access. Scholarship of Teaching and Learning in Psychology.

[B3-behavsci-15-01033] Bjork E. L., Bjork R. A., Gernsbacher M. A., Pew R. W., Hough L. M., Pomerantz J. R. (2011). Making things hard on yourself, but in a good way: Creating desirable difficulties to enhance learning. Psychology and the real world: Essays illustrating fundamental contributions to society.

[B4-behavsci-15-01033] Borman G. D., Choi Y., Hall G. J. (2021). The impacts of a brief middle-school self-affirmation intervention help propel African American and Latino students through high school. Journal of Educational Psychology.

[B5-behavsci-15-01033] Clark D. W., Briar D. W. (1993). What is ground water?.

[B6-behavsci-15-01033] Cohen G. L., Garcia J., Apfel N., Master A. (2006). Reducing the racial achievement gap: A social-psychological intervention. Science.

[B7-behavsci-15-01033] Cohen G. L., Garcia J., Purdie-Vaughns V., Apfel N., Brzustoski P. (2009). Recursive processes in self-affirmation: Intervening to close the minority achievement gap. Science.

[B8-behavsci-15-01033] Cortés Pascual A., Moyano Muñoz N., Quilez Robres A. (2019). The relationship between executive functions and academic performance in primary education: Review and meta-analysis. Frontiers in Psychology.

[B9-behavsci-15-01033] Covarrubias R., Herrmann S. D., Fryberg S. A. (2016). Affirming the interdependent self: Implications for latino student performance. Basic and Applied Social Psychology.

[B10-behavsci-15-01033] Covarrubias R., Valle I., Laiduc G., Azmitia M. (2019). “You never become fully independent”: Family roles and independence in first-generation college students. Journal of Adolescent Research.

[B11-behavsci-15-01033] Dowell N. M., McKay T. A., Perrett G. (2021). It’s not that you said it, it’s how you said it: Exploring the linguistic mechanisms underlying values affirmation interventions at scale. AERA Open.

[B12-behavsci-15-01033] Dunlosky J., Rawson K. A., Marsh E. J., Nathan M. J., Willingham D. T. (2013). Improving students’ learning with effective learning techniques: Promising directions from cognitive and educational psychology. Psychological Science in the Public Interest.

[B13-behavsci-15-01033] Gloria A. M., Kurpius S. E. R. (1996). The validation of the cultural congruity scale and the university environment scale with Chicano/a students. Hispanic Journal of Behavioral Sciences.

[B14-behavsci-15-01033] Goudeau S., Stephens N. M., Markus H. R., Darnon C., Croizet J.-C., Cimpian A. (2025). What causes social class disparities in education? The role of the mismatches between academic contexts and working-class socialization contexts and how the effects of these mismatches are explained. Psychological Review.

[B15-behavsci-15-01033] Gutchess A., Rajaram S. (2023). Consideration of culture in cognition: How we can enrich methodology and theory. Psychonomic Bulletin & Review.

[B16-behavsci-15-01033] Harackiewicz J. M., Canning E. A., Tibbetts Y., Priniski S. J., Hyde J. S. (2016). Closing achievement gaps with a utility-value intervention: Disentangling race and social class. Journal of Personality and Social Psychology.

[B17-behavsci-15-01033] Hartwig M. K., Dunlosky J. (2012). Study strategies of college students: Are self-testing and scheduling related to achievement?. Psychonomic Bulletin and& Review.

[B18-behavsci-15-01033] Hecht C. A., Priniski S. J., Tibbetts Y., Harackiewicz J. M. (2021). Affirming both independent and interdependent values improves achievement for all students and mitigates cultural mismatch for first-generation college students. Journal of Social Issues.

[B19-behavsci-15-01033] Hoshino-Browne E., Zanna A. S., Spencer S. J., Zanna M. P., Kitayama S., Lackenbauer S. (2005). On the cultural guises of cognitive dissonance: The case of easterners and westerners. Journal of Personality and Social Psychology.

[B20-behavsci-15-01033] Kornell N., Bjork R. A. (2007). The promise and perils of self-regulated study. Psychonomic Bulletin & Review.

[B21-behavsci-15-01033] Landis J. R., Koch G. G. (1977). The measurement of observer agreement for categorical data. Biometrics.

[B22-behavsci-15-01033] Miyake A., Kost-Smith L. E., Finkelstein N. D., Pollock S. J., Cohen G. L., Ito T. A. (2010). Reducing the gender achievement gap in college science: A classroom study of values affirmation. Science.

[B23-behavsci-15-01033] Pan S. C., Bjork R. A., Kahana M. J., Wagner A. D. (2024). Acquiring an accurate mental model of human learning: Toward an owner’s manual. Oxford handbook of memory.

[B24-behavsci-15-01033] Patronis M., Dubai A. (2014). The Effect of using the iPad on students’ performance in writing and reading comprehension: Pilot Study Report. Arab World English Journal.

[B25-behavsci-15-01033] Phillips L. T., Stephens N. M., Townsend S. S., Goudeau S. (2020). Access is not enough: Cultural mismatch persists to limit first-generation students’ opportunities for achievement throughout college. Journal of Personality and Social Psychology.

[B26-behavsci-15-01033] Rajaram S., Barber S., Roediger III H. L. (2024). Retrieval Processes in Memory. Learning and Memory: A Comprehensive Reference.

[B27-behavsci-15-01033] Schmader T., Johns M., Forbes C. (2008). An integrated process model of stereotype threat effects on performance. Psychological Review.

[B28-behavsci-15-01033] Shnabel N., Purdie-Vaughns V., Cook J. E., Garcia J., Cohen G. L. (2013). Demystifying values-affirmation interventions: Writing about social belonging is a key to buffering against identity threat. Personality and Social Psychology Bulletin.

[B29-behavsci-15-01033] Steele C., Berkowitz L. (1988). The psychology of self-affirmation: Sustaining the integrity of the self. Advances in Experimental Social Psychology.

[B30-behavsci-15-01033] Stephens N. M., Fryberg S. A., Markus H. R., Johnson C. S., Covarrubias R. (2012a). Unseen disadvantage: How American universities’ focus on independence undermines the academic performance of first-generation college students. Journal of Personality and Social Psychology.

[B31-behavsci-15-01033] Stephens N. M., Townsend S. S., Dittmann A. G. (2019). Social-class disparities in higher education and professional workplaces: The role of cultural mismatch. Current Directions in Psychological Science.

[B32-behavsci-15-01033] Stephens N. M., Townsend S. S., Markus H. R., Phillips L. T. (2012b). A cultural mismatch: Independent cultural norms produce greater increases in cortisol and more negative emotions among first-generation college students. Journal of Experimental Social Psychology.

[B33-behavsci-15-01033] Szmalec A., Verbruggen F., Vandierendonck A., Kemps E. (2011). Control of interference during working memory updating. Journal of Experimental Psychology: Human Perception and Performance.

[B34-behavsci-15-01033] Tibbetts Y., Harackiewicz J. M., Canning E. A., Boston J. S., Priniski S. J., Hyde J. S. (2016). Affirming independence: Exploring mechanisms underlying a values affirmation intervention for first-generation students. Journal of Personality and Social Psychology.

[B35-behavsci-15-01033] Tibbetts Y., Priniski S. J., Hecht C. A., Borman G. D., Harackiewicz J. M. (2018). Different institutions and different values: Exploring first-generation student fit at 2-year colleges. Frontiers in Psychology.

[B36-behavsci-15-01033] Valle I., Covarrubias R. (2024). Stories of conflict, agency, and hope: Master narratives underlying home-school mismatches for first-generation Latina students. Qualitative Psychology.

[B37-behavsci-15-01033] Vasquez-Salgado Y., Greenfield P. M., Burgos-Cienfuegos R. (2015). Exploring home-school value conflicts: Implications for academic achievement and well-being among Latino first-generation college students. Journal of Adolescent Research.

[B38-behavsci-15-01033] Wang Q. (2009). Are Asians forgetful? Perception, retention, and recall in episodic remembering. Cognition.

[B39-behavsci-15-01033] Wang Q. (2021). The cultural foundation of human memory. Annual Review of Psychology.

[B40-behavsci-15-01033] Wu Z., Spreckelsen T. F., Cohen G. L. (2021). A meta-analysis of the effect of values affirmation on academic achievement. Journal of Social Issues.

[B41-behavsci-15-01033] Yeager D. S., Walton G. M. (2011). Social-psychological interventions in education: They’re not magic. Review of Educational Research.

[B42-behavsci-15-01033] Yılmaz E., Phalet K., De Leersnyder J. (2024). Putting cultural mismatch theory to the test: Cultural fit of self-construal in predicting student outcomes. Journal of Social Issues.

